# The Association between Diabetes Mellitus and Keratoplasty in Poland in the Years 2013–2017

**DOI:** 10.3390/ijerph18189767

**Published:** 2021-09-16

**Authors:** Milena Kozioł, Michał Szymon Nowak, Monika Udziela, Jacek Paweł Szaflik

**Affiliations:** 1 Department of Analyses and Strategies, Ministry of Health, 00-952 Warsaw, Poland; m.koziol@mz.gov.pl; 2 Department of Ophthalmology, Public Ophthalmic Clinical Hospital (SPKSO), Medical University of Warsaw, 03-709 Warsaw, Poland; monika.udziela@wum.edu.pl (M.U.); szaflik@okulistyka.eu (J.P.S.); 3 Provisus Eye Clinic, 42-209 Czestochowa, Poland; 4 Saint Family Hospital Medical Center, 90-302 Lodz, Poland

**Keywords:** corneal transplantation, diabetes mellitus, urgent surgery

## Abstract

Background: The aim of this study is to assess the incidence and characteristic of corneal grafts and its association with diabetes mellitus in Poland in the years 2013–2017. Methods: The retrospective survey of the National Database of Hospitalizations was performed to identify all the corneal transplantations in Poland between January 2013 and December 2017. The comorbid diseases, in particular diabetes mellitus, were verified in the patients’ medical history. The logistic regression was applied to demonstrate the factors related to urgent surgeries. Results: In total, 5069 corneal grafts in 4710 patients were reported in the years 2013–2017. The number of CTs gradually increased by 37% from 914 (2.37 surgeries per one hundred thousand population) in the year 2013 to 1250 (3.25 surgeries per one hundred thousand population) in 2017, the final year of the study. CT incidence was the highest in subjects aged 70 years or older: 13.18 per one hundred thousand population in the year 2017. On average, about 22.43% of procedures were performed in patients with DM. The chance of urgent surgery was mostly correlated with full thickness CT and patients’ age. Conclusions: Despite the relatively low value of CT in Poland, there was an increasing number of CTs in the analyzed period.

## 1. Introduction

After cataract and glaucoma, corneal blindness is the third leading cause of blindness worldwide [1]. The prevalence of corneal blindness varies from country to country, depending on numerous factors, such as availability and general standards of eye care [2]. For instance, in some areas of Africa, even 90% of all blindness directly results from corneal pathology [3]. Unfortunately, in many cases, corneal transplantation (CT) is the only way to restore visual function when impairment caused by corneal damage results in an unacceptable quality of life [4]. A Global Survey of Corneal Transplantation and Eye Banking conducted in 2016, covering more than 95% of the world’s population, revealed a severe imbalance between the supply and demand for CT. Even though corneal transplantation is the most frequently performed type of transplant surgery worldwide, about 50% of the population has no access to it [5]. With an estimated 12.7 million people waiting for corneal transplantation in 2012, 1 in 70 of the needs are met worldwide. In Poland, corneal transplantation has been performed since 1926. CT is allowed only in public hospitals and is reimbursed entirely from public funds by the National Health Fund (NHF). Moreover, there are seven eye banks that are the only transplantation facilities to provide cornea tissue. In Poland, there is a shortage of donor corneas compared to recipients, which results in a long waiting time for corneal transplantation [6].

A systematic review shows that healthcare analysis often refers to comorbid factors that explain medical and systemic relations [7,8,9]. Several approaches have been developed to define the comorbid variables. One of the most popular methodologies has been proposed by Elixhauser et al. and it includes 30 comorbid factors. Among those factors, diabetes mellitus (DM) is a significant condition for the probability of death, hospital stay, and medical expenditure [10]. In terms of ocular disorders, DM is considered to be a serious condition that could affect the quality of vision and be an important risk factor for postoperative complications [11,12,13,14].

The primary aim of the present study is to analyze the incidence of corneal grafts and information related to the type of surgery, medical indications, and association with DM in Poland, during the period of 2013–2017. The secondary aim is to analyze the occurrence of comorbid factors (including DM) among graft recipients who had been waiting for corneal transplantations in Poland during 2013–2017.

## 2. Material and methods

### 2.1. Data Sources, Disease Codes, and Definitions

The present study was co-financed by the European Union funds through the European Social Fund under the Operational Program of Knowledge, Education, and Development and was a part of the Polish Ministry of Health project “Maps of Healthcare Needs —Database of Systemic and Implementation Analyses” [15,16,17].

The study design was a retrospective population-based survey. Assessed data from all patients who underwent CT in Poland between January 2013 and December 2017 were obtained from the national database of medical services [18]. In Poland, information related to all levels of healthcare services at public and private institutions financed from public sources is recorded in the database of the NHF. The information includes personal identification number (PESEL), medical data, and demography (date of birth, area code, sex of patients). The medical data include diagnoses coded according to the International Classification of Diseases, 10th Revision, and all performed procedures coded using the International Classification of Diseases, 9th Revision, ICD-9 procedure codes and unique NHF codes corresponding to certain hospital procedures. The population data about Poland were obtained from Central Statistical Office of Poland [19]. To define CT, the following NHF codes were used: B06, B07, and B08. The ICD-9 codes 11.63 and 11.64 were used to identify penetrating keratoplasty, and the ICD-9 codes 11.61 and 11.62 were used to identify lamellar keratoplasty. Keratoplasty was also considered with additional reported procedures (recognized with ICD-9 codes): 14.73, 14.74, and 14.75 corresponding to CT combined with vitrectomy; 12.59 and 12.69 corresponding to CT followed by glaucoma filtration surgery, and 13.71 corresponding to CT combined with cataract surgery. CTs reported in the years 2013–2017 were taken into consideration. The indications for CT were identified with ICD-10 codes reported to NHF after surgery, including ocular surface disease (H18.8), bullous keratopathy (H18.1), infectious keratitis and corneal ulcers (H16.0, H16.2, H16.3, H16.8, H16.9), keratoconus (H18.6), leucoma and corneal scars (H17.8, H17.9, H17.1), corneal pigmentations and deposits (H18.0), corneal dystrophies such as Fuchs’s dystrophy (H18.5), and band keratopathy (H18.4)—presented in Appendix A. 

During the study period, in the years 2013–2017, each patient reported in the NHF database with type 1 or type 2 diabetes mellitus (DM) was retrospectively identified with ICD-10 codes E10 and E11. If a patient had two different diagnoses, the more common ICD-10 code was taken into account. All diagnoses were then confirmed if a patient purchased antidiabetic drugs (and/or insulin) within the period of the study. Patients with the ICD-10 codes E12, E13, and E14 were excluded from the analysis. 

The investigation of comorbid diseases among patients who had been waiting for CT required access to the data collected by Poltransplant—an institution responsible for the transplantation process in Poland. Each patient who was active for at least one day on the waiting list for CT in the Poltransplant database was included in the study. Personal National identification number (PESEL) allowed the Poltransplant records to be merged with NHF data in order to verify the 2-year medical history of each patient. Medical history was described by 30 variables defined by ICD-10 codes presented in Appendix B.

### 2.2. Statistical Analysis

Statistical analyses included the descriptive statistics of corneal graft incidence in the years 2013–2017 in Poland matched with the general Polish population and the patients’ medical and demographical features. In addition, the occurrence of comorbid diseases (including DM) among persons who waited for CT in the years 2013–2017 has been verified. A logistic regression model has been built to evaluate the factors correlated with urgent admission to hospital for CT in Poland during 2013–2017. The independent variable had a binary distribution (1—urgent, 0—elective surgery). The results of model estimation were presented in terms of odds ratio (OR) with a 95% confidence interval (CI). The significance of parameter estimation was validated with a *p*-value < 0.05. R statistical software V. 3.5.2 was used for all analyses.

The study adhered to the tenets of the Declaration of Helsinki for research involving human subjects and the study protocol was approved by the Polish Ministry of Health, which is authorized by the law of the Republic of Poland to process the National Health Fund data.

## 3. Results

**Figure 1:**
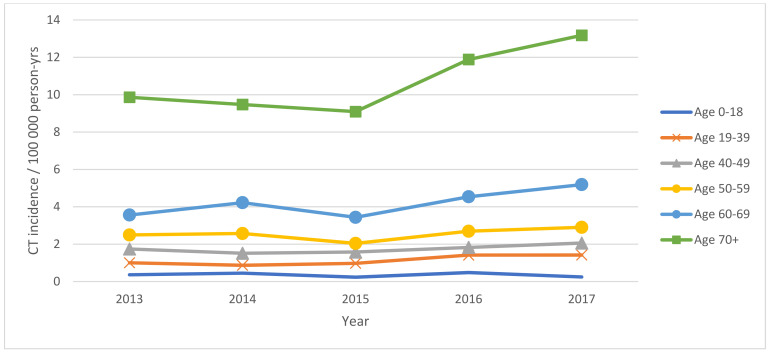
Incidence of corneal grafts in age groups in Poland during 2013–2017.

**Figure 2:**
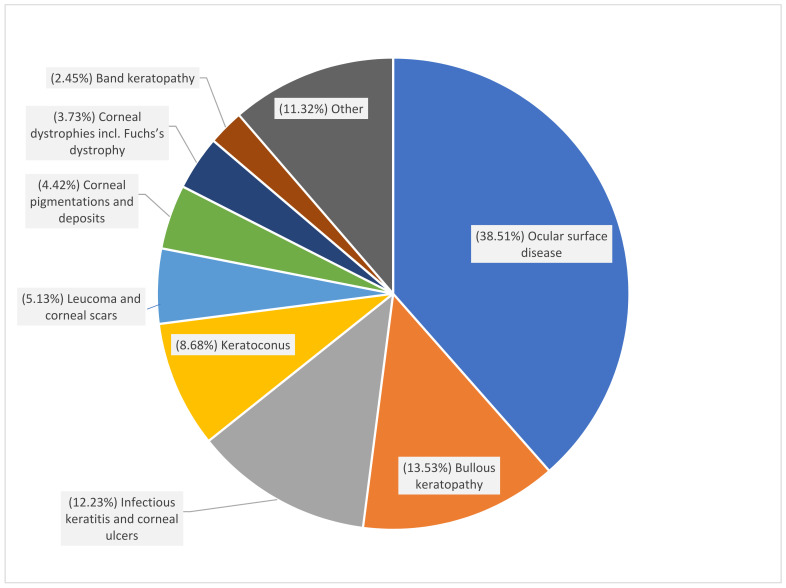
Indications for corneal grafts in Poland in the years 2013–2017.

**Figure 3:**
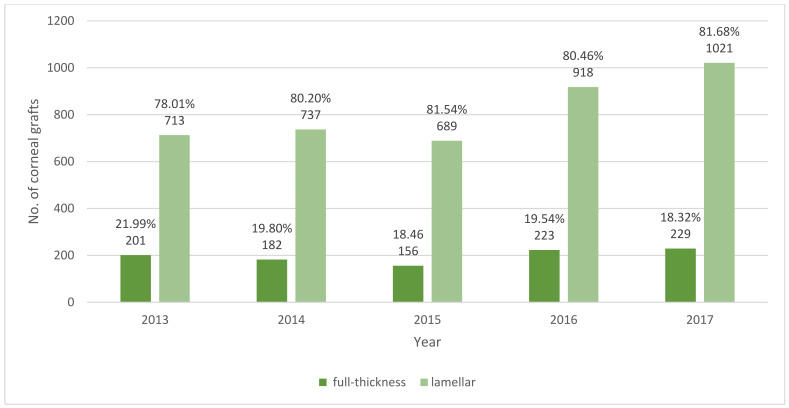
Number of full thickness and lamellar corneal grafts in Poland during 2013–2017.

**Figure 4:**
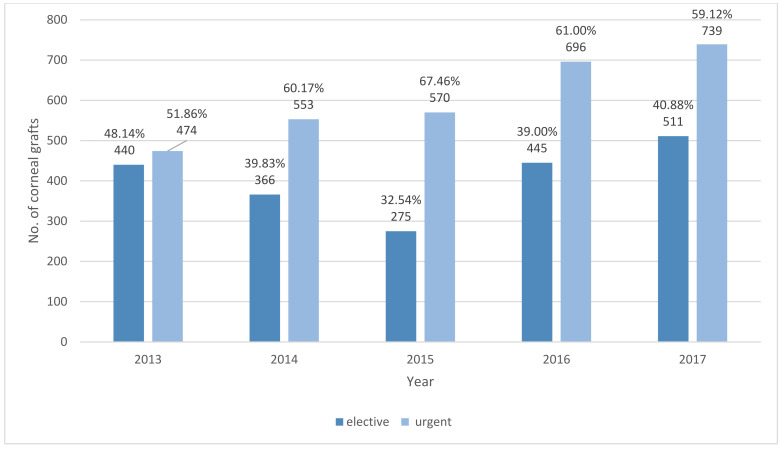
Number of elective and urgent corneal grafts in Poland during 2013–2017.

In total, 5069 corneal transplantations in 4710 patients were reported in Poland during 2013–2017 (Figure 1, Table 1 and Table 2). The number of CTs gradually increased from 914 (2.37 surgeries per one hundred thousand population) in the year 2013 to 1250 (3.25 surgeries per one hundred thousand population) in 2017, the final year of the study. CT incidence was the highest in subjects aged 70 years or older: 13.18 per one hundred thousand population in the year 2017. On average, about 22.43% of the procedures were performed in patients with DM. The demographic characteristics of all the patients who underwent CT showed that 49.15% of the group were male, the mean age of the study subjects at the time of CT was 61.63 ± 19.20 years, and 69.94% of them lived or had lived in urban areas. The indications for corneal grafts in Poland in the years 2013–2017 are presented in Figure 2 and Table 3. The most frequent indications reported to the NHF database were ocular surface disease (38.51%), followed by bullous keratopathy (13.53%), infectious keratitis, and corneal ulcers (12.23%). Among the persons with DM who underwent CT in Poland during 2013–2017, ocular surface disease was the most common indication for CT, present in 39.18 and 41.77% of patients with type 2 DM and type 1 DM, respectively. The clinical characteristics of corneal grafts in Poland are presented in Table 4, Figure 3 and Figure 4. During the study period, 19.55% of CTs were related to full-thickness and 80.45% of CTs were related to lamellar corneal grafts in Poland. This ratio was stable over the years 2013–2017. Twenty-one percent of all the CTs were combined with other ophthalmic procedures, including 997 (19.39%) combined with cataract surgery, 27 (0.52%) combined with glaucoma surgery, and 74 (1.44%) with pars plana vitrectomy (PPV), respectively.

The logistic regression model showed (Table 5) that full-thickness corneal graft was a significant factor correlated with urgent admission (OR = 11.54, lamellar corneal grafts were a reference group). In comparison with the patients aged 0–18, the odds ratios for all the other age groups were reduced and statistically significant. In addition, information about CT combined with other ophthalmic procedures occurred as an important variable in the model. The chance of urgent admission in that group was 18% lower compared to the reference group. The DM, sex, and residence factors did not significantly influence urgent hospital admission for CT. The ROC of the presented model was 0.7.

Table 6 demonstrates the characteristics of the population waiting for CT in terms of comorbid factors in the years 2013–2017 in Poland. The selected variables defined by the occurrence of suitable ICD-10 codes in the medical history of the patients accurately describe the general health condition of the persons waiting for CT. It turns out that 19.63% of the group were reported with type 2 DM and 1.31% with type 1 DM. That implies that the prevalence of DM in the group was about three times higher than in the general population.

## 4. Discussion

This study showed, for the first time, the incidence and characteristics of corneal grafts including information related to the type of surgery, medical indications, and correlation with DM in Poland, during 2013–2017. Until now, there have been no studies analyzing the epidemiologic indicators of CT based on the nationwide population from Central and Eastern Europe. The mean age of the study group was over 60 years, which could indicate the late diagnosis of corneal diseases in Poland [20,21,22,23,24]. 

The United States of America, with 19.91 transplants per one hundred thousand people, had the highest rate of CTs in the world. In Europe, the highest score was identified in The Netherlands and Switzerland, with about 8.80 per one hundred thousand people [5]. In Poland, the score was about 2.37 transplantations per one hundred thousand people in 2013 and this increased to 3.25 procedures per one hundred thousand in 2017. Compared to countries with a similar gross domestic product (GDP) per capita, such as the Czech Republic or Portugal, Poland still has a low value of both CT and eye banking [5]. Poland is a middle-income and relatively conservative Catholic country, which could be an important reason for a low number of CTs compared to other countries in Europe. However, a positive trend was observed—the number of CTs increased by 37% during the study period in Poland. This was a relatively high score when compared to trends in other European countries. For instance, in Germany there was only a 25% increase during the period 2012–2016 [22].

The indications for CT are different across countries and depend on the sociodemographic conditions of each population. In Poland, the main four indications accounting for 70% of CTs were ocular surface disease (38.51%), bullous keratopathy (13.53%), infectious keratitis and corneal ulcers (12.23%), and keratoconus (8.68%). In Germany [22], the most frequent indication for CTs was Fuchs endothelial corneal dystrophy (46%), followed by bullous keratopathy and keratoconus. Similarly, in Great Britain, keratoconus (15%), Fuchs endothelial corneal dystrophy (9.3%), and bullus keratopathy (7.6%) were the most often reported indications for CT [22,24]. 

During the study period, the general proportion of full-thickness and lamellar CTs was constant in Poland. In contrast, in a 10-year Canadian study, a significant decrease in the number of corneal transplants performed with penetrating keratoplasty had been observed [25]. Moreover, a Germany study revealed a shift from penetrating to lamellar procedures from 4% in 2006 to 60% in 2016 as well [22]. The indications for full-thickness and lamellar transplantation also differed in Poland, i.e., 41.86% of all the full-thickness CTs was related to ocular surface disease, while the most frequent indication for lamellar CTs was bullous keratopathy (30.27%). 

Diabetes mellitus seems to have a meaningful impact on vision, mostly because of diabetic retinopathy and diabetic cataract [26]. At the same time, the cornea suffers from diabetic complications. According to Russian authors [27], up to 70% of DM patients could be affected by corneal problems. The results of other previously published studies revealed that DM is considered a preoperative risk factor of graft failure [28]. In Poland, about 22% of the persons who underwent CT suffered from DM, which is three times more than the prevalence of DM in the Polish population [17]. The differences could be attributed to the age distribution of the study group. However, it was shown that the occurrence of DM was several times higher in the young age group within the study group than in the overall population. Meanwhile, the highest occurrence of both CT and DM refers to the oldest age group (over 30% of the patients with CT were reported with DM in this group). DM occurrence differs between the indications for corneal graft in Poland. Relatively low values of the occurrence of DM were observed in the patients with keratoconus. 

In Poland, CT may be performed as either an elective or urgent procedure in a hospital. Our analysis revealed that the chance of urgent surgery is mostly correlated with patient age, CT not combined with other procedures, and penetrating CT. The chance of urgent hospitalization is about two times higher for children compared to adults. That result is strongly correlated with the rules of the waiting list in Poland where children are on the priority list [6]. The chance of lamellar keratoplasty performed urgently is over 11 times lower than that of full-thickness CT. Other factors, including DM and sex, were revealed to be statistically non-significant in multiple regression analysis. Although the popularity of lamellar CT is rising worldwide, the procedure is more challenging and requires an experienced surgeon [29]. An analysis of comorbid factors among the persons on the waiting list for CT in Poland reveals 1.31% of subjects with type 1 DM and 19.63% with type 2 DM. This is a high score compared to DM prevalence in the overall Polish population but correlates with the age distribution of the patients on the waiting list. Furthermore, diseases related to depression and mental disorders could be highlighted. According to the Institute for Health Metrics and Evaluation [30], the prevalence of depression among the persons on the waiting list was over two times higher than in the general population in Poland.

The limitations of the present study include a lack of pathologic reports, laterality, and information of the number of anterior and posterior lamellar grafts in both the NHF and Poltransplant databases. Therefore, we were unable to recognize re-graft cases and investigate the potential risk factors of CT failures. However, this probably only had a minor impact on the study findings. Population size, national recruitment, and the impact of its findings on public healthcare policy are the most important strengths of the present study. 

## 5. Conclusions

In conclusion, this study reported the incidence and characteristics of corneal transplantation in Poland during 2013–2017. Despite the relatively low value of both CT and eye banking in Poland, there was an increasing number of CTs in the analyzed period. However, the statistical analysis did not indicate DM as a significant factor of urgent CT, it was shown that about 23% of all CTs were performed on persons with DM. We believe this epidemiological study will help clinicians and healthcare institutions in Poland to better manage and treat patients with indications for CT. 

## Figures and Tables

**Table 1:** Incidence of corneal grafts in Poland during 2013–2017.

	2013	2014	2015	2016	2017	All (Average)
Age 0–18	7,431,731	7,367,066	7,309,001	7,286,480	7,299,996	7,338,855
DM in the subgroup, %	0.18%	0.19%	0.20%	0.21%	0.22%	0.20%
No. of corneal grafts	27	33	17	35	18	130
Incidence/100,000 person-yrs	0.36	0.45	0.23	0.48	0.25	0.35
DM, %	0.00%	0.00%	0.00%	2.86%	0.00%	0.57%
Age 19–39	12,355,235	12,201,430	12,015,345	11,799,562	11,570,164	11,988,347
DM in the subgroup, %	0.58%	0.68%	0.79%	0.90%	1.01%	0.79%
fNo. of corneal grafts	124	106	117	167	164	678
Incidence/100,000 person-yrs	1.00	0.87	0.97	1.42	1.42	1.14
DM, %	4.03%	4.72%	1.71%	1.20%	1.83%	2.70%
Age 40–49	4,879,816	4,956,005	5,064,587	5,202,444	5,341,530	5,088,876
DM in the subgroup, %	2.14%	2.30%	2.45%	2.59%	2.70%	2.44%
No. of corneal grafts	85	75	80	95	110	445
Incidence/100,000 person-yrs	1.74	1.51	1.58	1.83	2.06	1.74
DM, %	10.59%	8.00%	7.50%	16.84%	7.27%	10.04%
Age 50–59	5,536,118	5,406,320	5,245,352	5,089,290	4,928,276	5,241,071
DM in the subgroup, %	6.87%	7.16%	7.38%	7.57%	7.68%	7.33%
No. of corneal grafts	138	139	107	137	143	664
Incidence/100,000 person-yrs	2.49	2.57	2.04	2.69	2.90	2.54
DM, %	15.22%	15.83%	10.28%	10.22%	9.79%	12.27%
Age 60–69	4,409,809	4,642,821	4,888,294	5,024,702	5,127,315	4,818,588
DM in the subgroup, %	15.53%	16.20%	16.76%	17.15%	17.34%	16.60%
No. of corneal grafts	157	196	168	228	266	1015
Incidence/100,000 person-yrs	3.56	4.22	3.44	4.54	5.19	4.19
DM, %	35.67%	26.02%	36.31%	25.88%	24.06%	29.59%
Age 70+	3,882,950	3,904,960	3,914,660	4,030,514	4,166,277	3,979,872
DM in the subgroup, %	21.41%	22.88%	24.15%	25.27%	26.17%	23.98%
No. of corneal grafts	383	370	356	479	549	2137
Incidence/100,000 person-yrs	9.86	9.48	9.09	11.88	13.18	10.70
DM, %	31.85%	32.16%	35.39%	33.19%	30.24%	32.57%
All	38,495,659	38,478,602	38,437,239	38,432,992	38,433,558	38,455,610
DM in the subgroup, %	5.42%	5.83%	6.20%	6.56%	6.86%	6.17%
No. of corneal grafts	914	919	845	1141	1250	5069
Incidence/100,000 person-yrs	2.37	2.39	2.20	2.97	3.25	2.64
DM, %	23.30%	22.09%	24.38%	22.00%	20.40%	22.43%

**Table 2:** Demographic characteristics of patients with corneal grafts in Poland during 2013–2017.

	2013	2014	2015	2016	2017	All
Mean age ± SE	60.99 ± 19.45	61 ± 18.8	61.25 ± 19.32	61.29 ± 20.04	63.15 ± 18.4	61.63 ± 19.2
Women (%)	426 (49.71%)	412 (47.96%)	388 (49.87%)	554 (52.31%)	615 (53.15%)	2395 (50.85%)
Men (%)	431 (50.29%)	447 (52.04%)	390 (50.13%)	505 (47.69%)	542 (46.85%)	2315 (49.15%)
Urban residence (%)	612 (71.41%)	600 (69.85%)	550 (70.69%)	740 (69.88%)	792 (68.45%)	3294 (69.94%)
Rural Residence (%)	245 (28.59%)	259 (30.15%)	228 (29.31%)	319 (30.12%)	365 (31.55%)	1416 (30.06%)

**Table 3:** Indications for corneal grafts in Poland in the years 2013–2017.

Indication	No. of Corneal Grafts 2013–2017 (% of all)	No. of Subjects with Corneal Grafts and Type 1 DM (% of All)	No. of Subjects with Corneal Grafts and Type 2 DM (% of All)	No. of Full-Thickness Corneal Grafts 2013–2017 (% of All)	No. of Partial-Corneal Grafts 2013–2017 (% of All)
Ocular surface disease	1952 (38.51%)	33 (41.77%)	411 (39.18%)	1707 (41.86%)	245 (24.72%)
Bullous keratopathy	686 (13.53%)	9 (11.39%)	182 (17.35%)	386 (9.47%)	300 (30.27%)
Infectious keratitis and corneal ulcers	620 (12.23%)	19 (24.05%)	131 (12.49%)	591 (14.49%)	29 (2.93%)
Keratoconus	440 (8.68%)	1 (1.27%)	21 (2.00%)	386 (9.47%)	54 (5.45%)
Leucoma and corneal scars	260 (5.13%)	3 (3.80%)	53 (5.05%)	242 (5.93%)	18 (1.82%)
Corneal pigmentations and deposits	224 (4.42%)	0 (0.00%)	59 (5.62%)	194 (4.76%)	30 (3.03%)
Corneal dystrophies incl. Fuchs’s dystrophy	189 (3.73%)	2 (2.53%)	38 (3.62%)	39 (0.96%)	150 (15.14%)
Band keratopathy	124 (2.45%)	2 (2.53%)	26 (2.48%)	80 (1.96%)	44 (4.44%)
Other	574 (11.32%)	10 (12.66%)	128 (12.20%)	453 (11.11%)	121 (12.21%)
All	5069 (100%)	79 (100%)	1049 (100%)	991 (100%)	4078 (100%)

**Table 4:** Clinical characteristics of corneal grafts in Poland from 2013 to 2017.

	2013	2014	2015	2016	2017	All
No. of full-thickness corneal grafts (%)	201 (21.99%)	182 (19.8%)	156 (18.46%)	223 (19.54%)	229 (18.32%)	991 (19.55%)
No. of lamellar corneal grafts (%)	713 (78.01%)	737 (80.2%)	689 (81.54%)	918 (80.46%)	1021 (81.68%)	4078 (80.45%)
No. of corneal grafts combined with cataract surgery (%)	187 (20.46%)	193 (21%)	160 (18.93%)	214 (18.76%)	220 (17.6%)	997 (19.39%)
No. corneal grafts combined with glaucoma surgery (%)	5 (0.55%)	9 (0.98%)	5 (0.59%)	3 (0.26%)	5 (0.4%)	27 (0.52%)
No. of corneal grafts combined with PPV (%)	14 (1.53%)	10 (1.09%)	14 (1.66%)	9 (0.79%)	27 (2.16%)	74 (1.44%)
No. of corneal grafts in urgent admission (%)	474 (51.86%)	553 (60.17%)	570 (67.46%)	696 (61.00%)	739 (59.12%)	3032 (59.81%)

**Table 5:** A logistic regression model evaluating the factors correlated with urgent vs. elective CT in Poland during 2013–2017.

	OR	95% CI	*p*-Value
DM type (no)	reference		
Type 1 DM	1.01	(0.63; 1.64)	0.976
Type 2 DM	1.05	(0.9; 1.23)	0.498
Type—partial	reference		
Type—full	11.54	(10.08; 18.58)	0.000 *
Age 0–18	reference		
Age 19–39	0.45	(0.27; 0.72)	0.001 *
Age 40–49	0.49	(0.29; 0.79)	0.004 *
Age 50–59	0.49	(0.3; 0.78)	0.004 *
Age 60–69	0.44	(0.27; 0.69)	0.001 *
Age 70+	0.35	(0.21; 0.54)	0.000 *
Combined (no)	reference		
Combined (yes)	0.82	(0.70; 0.96)	0.011 *
Sex (man)	reference		
Sex (woman)	1	(0.88; 1.13)	0.959
Residence (rural)	reference		
Residence (urban)	0.91	(0.8; 1.04)	0.168

* statistical significance.

**Table 6:** Characteristics of the population waiting for keratoplasty in terms of comorbid factors in the years 2013–2017 in Poland.

Comorbidity Variable	No. of People on the Waiting List	Comorbidity Variable	No. of People on the Waiting List
Age	62.05 ± 19.00	Fluid and electrolyte disorders	176 (2.22%)
Sex (women)	4144 (52.30%)	AIDS/HIV	0 (0.00%)
Urban	5466 (69.10%)	Hypothyroidism	809 (10.21%)
Alcohol abuse	206 (2.60%)	Hypertension	4770 (60.21%)
Blood loss anaemia	21 (0.27%)	Liver disease	220 (2.78%)
Arrhythmias	1436 (18.13%)	Lymphoma	42 (0.53%)
Solid tumor without metastasis	654 (8.26%)	Metastatic cancer	53 (0.67%)
Congestive heart failure	1297 (16.37%)	Other neurological disorders	352 (4.44%)
Coagulopathy	76 (0.96%)	Obesity	210 (2.65%)
Chronic pulmonary disease	1241 (15.67%)	Paralysis	69 (0.87%)
Deficiency anaemia	169 (2.13%)	Pulmonary circulation disorders	81 (1.02%)
Type 1 Diabetes	104 (1.31%)	Psychoses	91 (1.15%)
Type 2 Diabetes	1555 (19.63%)	Peptic ulcer disease excluding bleeding	52 (0.66%)
Depression	613 (7.74%)	Peripheral vascular disorders	1335 (16.85%)
Drug abuse	47 (0.59%)	Renal failure	422 (5.33%)
Fluid and electrolyte disorders	176 (2.22%)	Rheumatoid arthritis/ collagen vascular diseases	460 (5.81%)
AIDS/HIV	0 (0.00%)	Valvular disease	356 (4.49%)
Hypothyroidism	809 (10.21%)	Weight loss	45 (0.57%)

## Data Availability

The National Health Fund Registry data are available at http://www.nfz.gov.pl (accessed on 10 August 2020), and the Statistics Poland data are available at http://www.stat.gov.pl (accessed on 10 August 2020).

## Appendix A

**Table A1:** Indications for CTs in Poland during 2013–2017.

Indication	Indication ICD-10
Ocular surface diseases	H18.8
Bullous keratopathy	H18.1
Infectious keratitis and corneal ulcers	H16.0, H16.2, H16.3, H16.8, H16.9
Keratoconus	H18.6
Leucoma and corneal scars	H17.8, H17.9, H17.1
Corneal pigmentations and deposits	H18.0
Corneal dystrophies incl. Fuchs’s dystrophy	H18.5
Band keratopathy	H18.4
Other	-

## Appendix B

**Table A2:** Medical history of subjects waited for CTs in Poland during 2013–2017.

	Comorbidity Variable	Comorbidity Group (ICD-10 Codes)
1	AIDS/HIV	B20, B21, B22, B24
2	Alcohol abuse	F10, E52, G62.1, I42.6, K29.2, K70.0, K70.3, K70.9, T51, Z50.2, Z71.4, Z72.1
3	Blood loss anemia	D50.0
4	Arrhythmias	I44.1, I44.2, I44.3, I45.6, I45.9, I47–I49, R00.0, R00.1, R00.8, T82.1, Z45.0, Z95.0
5	Chronic pulmonary disease	I27.8, I27.9, J40–J47, J60–J67, J68.4, J70.1, J70.3
6	Coagulopathy	D65, D66, D67, D68, D69.1, D69.3, D69.4, D69.5, D69.6
7	Congestive heart failure	I09.9, I11.0, I13.0, I13.2, I25.5, I42.0, I42.5, I42.6, I42.7, I42.8, I42.9, I43, I50, P29.0
8	Deficiency anemia	D50.8, D50.9, D51, D52, D53
9	Depression	F20.4, F31.3, F31.4, F31.5, F32, F33, F34.1, F41.2, F43.2
10	Diabetes, Type 1	E10 confirmed with purchased medications
11	Diabetes, Type 2	E11 confirmed with purchased medications
12	Drug abuse	F11, F12, F13, F14, F15, F16, F18, F19, Z71.5, Z72.2
13	Fluid and electrolyte disorders	E22.2, E86, E87
14	Hypertension	I10, I11, I12, I13, I15
15	Hypothyroidism	E00, E01, E02, E03, E89.0
16	Liver disease	B18, I85, I86.4, I98.2, K70, K71.1, K71.3–K71.5, K71.7, K72–K74, K76.0, K76.2–K76.9, Z94.4
17	Lymphoma	C81, C82, C83, C84, C85, C88, C96, C90.0, C90.2
18	Metastatic cancer	C77, C78, C79, C80
19	Obesity	E66
20	Other neurological disorders	G10–G13, G20–G22, G25.4, G25.5, G31.2, G31.8, G31.9, G32, G35–G37, G40, G41, G93.1, G93.4, R47.0, R56
21	Paralysis	G04.1, G11.4, G80.1, G80.2, G81, G82, G83.0, G83.1, G83.2, G83.3, G83.4, G83.9
22	Peptic ulcer disease excluding bleeding	K25.7, K25.9, K26.7, K26.9, K27.7, K27.9, K28.7, K28.9
23	Peripheral vascular disorders	I70, I71, I73.1, I73.8, I73.9, I77.1, I79.0, I79.2, K55.1, K55.8, K55.9, K95.8, Z95.9
24	Psychoses	F20, F22, F23, F24, F25, F28, F29, F30.2, F31.2, F31.5
25	Pulmonary circulation disorders	I26, I27, I28.0, I28.8, I28.9
26	Renal failure	I12.0, I13.1, N18, N19, N25.0, Z49.0, Z49.1, Z49.2, Z94.0, Z99.2
27	Rheumatoid arthritis/collagen vascular diseases	L94.0, L94.1, L94.3, M05, M06, M08, M12.0, M12.3, M30, M31.0–M31.3, M32–M35, M45, M46.1, M46.8, M46.9
28	Solid tumor without metastasis	C00–C26, C30–C34, C37–C41, C43, C45–C58, C60–C76, C97
29	Valvular disease	A52.0, I05–I08, I09.1, I09.8, I34–I39, Q23.0–Q23.3, Z95.2, Z95.3, Z95.4
30	Weight loss	E40–E46, R63.4, R64
